# Prevalence of the Use of Oral Contraceptives and Hormone Replacement Therapy in Japan: The Japan Nurses’ Health Study

**DOI:** 10.2188/jea.JE20200207

**Published:** 2022-03-05

**Authors:** Toshiyuki Yasui, Yuki Ideno, Hiromitsu Shinozaki, Yoshikazu Kitahara, Kazue Nagai, Kunihiko Hayashi

**Affiliations:** 1 Department of Reproductive and Menopausal Medicine, Institute of Biomedical Sciences, Tokushima University Graduate School, Tokushima, Japan; 2 Center for Mathematics and Data Science, Gunma University, Maebashi, Japan; 3 Graduate School of Health Sciences, Gunma University, Maebashi, Japan; 4 Department of Obstetrics and Gynecology, Gunma University Graduate School of Medicine, Maebashi, Japan

**Keywords:** hormone replacement therapy, oral contraceptive, prevalence, nurse

## Abstract

**Background:**

There have been few community-based epidemiological studies in which the prevalence of exogenous hormone use, including the use of oral contraceptives (OCs) and hormone replacement therapy (HRT), has been accurately assessed in Japan.

**Methods:**

We have been conducting repeated surveys of participants in the Japan Nurses’ Health Study (JNHS), as a nationwide prospective cohort study, since 2001. We determined the prevalence of exogenous hormone use at baseline and during a 10-year follow-up period. A total of 15,019 female nurses participated in the JNHS follow-up cohort. We determined the prevalence of OC use in 14,839 women <60 years of age at baseline and the prevalence of HRT use in 7,915 women, excluding premenopausal women, at the last time they answered a questionnaire. The duration of HRT use was estimated using the Kaplan-Meier method.

**Results:**

Six percent of the participants used OCs. The proportion of HRT users who stopped HRT before the baseline survey, the proportion of women using HRT during the follow-up period, and the proportion of all of the participants who had used HRT were 3.2%, 10.6%, and 13.8%, respectively. The median duration of HRT use was 2 years.

**Conclusions:**

The lifetime prevalences of exogenous hormone use during this prospective study conducted in Japanese nurses were 6.0% for OCs and 13.8% for HRT. The information obtained in this study will be useful for clarification of the association between exogenous estrogen exposure and estrogen-related diseases as future research.

## INTRODUCTION

In women of reproductive age, oral contraceptives (OCs) are the most effective and widely used forms of contraception. Indeed, in the United States, more than 80% of sexually active women aged 15–44 years have reported using OCs.^1^ A survey conducted by the Japan Family Planning Association showed that the prevalence of OC use in Japan in 2004 was 3.0%.^2^ However, there have been few epidemiological studies in which the extent of OC use was assessed in a longitudinal study in Japan.

Hormone replacement therapy (HRT) has an important role in the management of menopausal symptoms and osteoporosis. According to the Women’s Health Initiative (WHI) reports in 2002 and 2004, no preventive effects of HRT on cardiovascular diseases were found.^3^^,^^4^ In the United States, it was reported that the annual proportion of women aged 50–74 years who were on HRT declined from 42% in 2001 to 28% in 2003.^5^ The proportion of women undergoing HRT also decreased in 17 European countries.^6^ However, the timing hypothesis for benefits and risks of HRT showed that women younger than 60 years of age or within 10 years after menopause with menopausal symptoms have many benefits of HRT and few risks from HRT.^7^ Thereafter, the effects of the route of administration and the dosages and types of estrogen and progestogen were investigated, and it was suggested that a personalized approach should be used for therapy.^8^

There have been many studies on the prevalence of HRT in which the prevalence was assessed at a single time point. The estimated proportions of current users of HRT in women aged 45–69 years were 13.2% in Finland, 5.3% in Sweden, and 9.7% in Belgium in 2013.^9^ It was also reported that 11.8% of menopausal women in Australia were current users of HRT at the time of a survey conducted in 2010.^10^ In Japan, a cross-sectional survey conducted in Takayama City in 1992 showed that only 9.3% of women aged 45–64 years had ever used HRT and that only 2.5% of women were current users of HRT at the time of the survey.^11^ However, there have been few community-based epidemiological studies in which HRT use was assessed in a longitudinal study, except for some prospective cohort studies (eg, the Study of Women’s Health Across the Nation, the Nurses’ Health Study and the Danish Nurses Cohort Study).^12^^–^^15^

The prevalences of exogenous hormone use, including the use of OCs and HRT, in previous studies differed depending on the study design. The prevalence at a single time point in a cross-sectional study may differ from that during a longitudinal study because steady state prevalence depends not only on the incidence of commencing drug use but also on the mean duration of use.^16^ The prevalence of drug use determined in a longitudinal study is likely to be closer to the true lifetime prevalence.

Simultaneous assessments of the prevalence of OC use and the prevalence of HRT use would provide important epidemiological and clinical information for women during their lifetime. Determination of the total amount of OC use and total amount of HRT use in various life stages is necessary to examine the effects of cumulative exposure to exogenous estrogen in a women’s lifetime, although ages, reasons for use and durations of use are different for OCs and HRT.

We assessed the prevalence of exogenous reproductive hormone use, including OC use and HRT use, as the lifetime prevalence in Japan before the baseline survey and during a 10-year follow-up period that was based on biennial surveys of the Japan Nurses’ Health study (JNHS) population, as a large prospective cohort study. We also estimated the cumulative incidence of HRT use using the Kaplan-Meier method. The age at which HRT use commenced, the duration of HRT use, and the HRT administration route were also investigated.

## METHODS

### Data collection and study participants

The JNHS was a large prospective cohort study designed to investigate the effects of lifestyle and healthcare practices on the health of Japanese women. The details of the study design have been previously described.^17^ A cross-sectional baseline survey was conducted between 2001 and 2007. The target population was working women who were 21 years of age or older and lived in Japan at the time of the baseline survey, and the source population was nurses, public health nurses and midwives. Each applicant was mailed a self-administered questionnaire regarding their basic demographic characteristics; lifestyle habits; physical condition; reproductive history, including questions related to menstrual status, such as menstruation cycle, existence of menopause, day of last menstruation, and cause of menopause; and use of hormonal agents, together with a list of female hormonal drugs with pictures.

A total of 49,927 female nurses participated in the JNHS cross-sectional survey. Of the 49,927 women, 15,019 women agreed to participation in the follow-up survey. In the baseline survey and biennially during the follow-up study, we asked for information on menopausal status, age at menopause, cause of menopause (natural menopause, surgical menopause, menopause secondary to radiation therapy or chemotherapy, or any other cause), and any past history of unilateral/bilateral oophorectomy or hysterectomy. Since age at menopause was estimated using the Kaplan-Meier method to be 54 years in 90% of the women who had undergone natural menopause in the JNHS population,^18^ the age at menopause was set to 54 years for women who underwent hysterectomy.

### Identification of OC and HRT users

Previous use of female hormone preparations was ascertained before and after menopause (never used, had used before the baseline survey, or currently using). In the baseline survey, the participants identified the specific hormone preparation using the list and pictures of drugs. With regard to OCs, we asked about the use of exogenous female hormones other than those used in HRT before the baseline survey and during the 10-year study period. Three gynecologists used the specific drug name regardless of the purpose and identified OC users, considering that there are various purposes for using exogenous female hormones other than HRT, such as treatment for infertility, functional uterine bleeding, and dysmenorrhea, as well as contraception. With regard to HRT, the duration of HRT use, the type of HRT, and the pattern of progestogen use were ascertained. The three gynecologists identified HRT users via items such as the age they took hormones, the purposes of the hormone use, and their symptoms, as well as the type of drug and the specific drug name.

The Ethics Committee of Gunma University reviewed and approved the study (#101, 2001 and #18-11, 2007). Written informed consent was obtained from all participants.

### Statistical analysis

**Figure 1.**
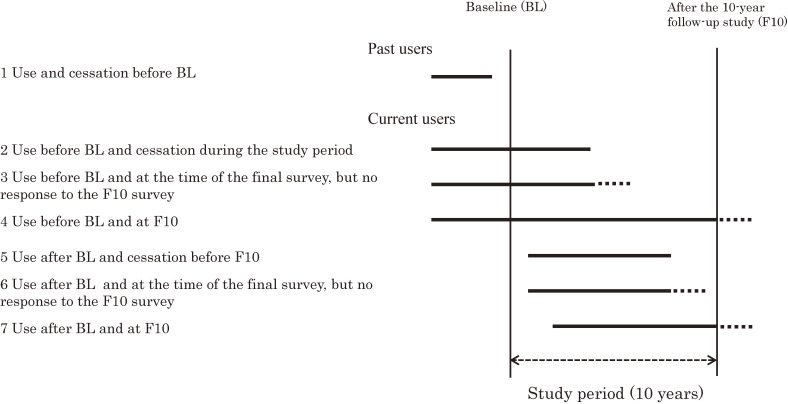
Classification of HRT users. BL, baseline; F10, after the 10-year follow-up study; HRT, hormone replacement therapy; OC, oral contraceptive.

A total of 15,019 female nurses participated in the JNHS cohort. With respect to OC use, data for 14,839 women who were less than 60 years of age in the baseline survey, an age range in which OCs might be used, were analyzed. We determined the sum of OC use including any past use as the lifetime prevalence of OC use. Data for 7,915 women, excluding premenopausal women with regular menstruation at the last time point of the longitudinal study who did not need treatment for menopausal symptoms, were analyzed to determine the prevalence of HRT use. HRT users were divided into the following seven groups for analysis: 1) women who had used HRT and had stopped HRT before the baseline survey, 2) women who had used HRT before the baseline survey and stopped HRT during the longitudinal study period, 3) women who had used HRT before the baseline survey and were using HRT at the last time they answered a questionnaire during the 10-year follow-up survey, 4) women who had used HRT before the baseline survey and were using HRT at the time of the 10-year survey, 5) women who started HRT after the baseline survey and stopped HRT before the 10-year survey, 6) women who started HRT after the baseline survey and were using HRT at the last time they answered a questionnaire and did not respond to the 10-year survey, and 7) women who started HRT after the baseline survey and were using HRT at the time of the 10-year survey (Figure 1). The HRT users included women who had stopped using exogenous hormone preparations before the baseline survey and had never used HRT again during the survey period (past users) and women who had used exogenous hormone preparations at least once since the baseline survey (current users). According to the classification of HRT users, we calculated the simple prevalence of HRT use to estimate lifetime prevalence. Furthermore, the cumulative incidence of HRT use at the age of 60 years was estimated using the Kaplan-Meier method.

In women who had used hormonal preparations but with interruptions in their use, the duration of HRT was defined as the sum of the durations of HRT use. We estimated the median duration of HRT use via the Kaplan-Meier method to consider censored data. We also categorized the hormonal preparations according to their route of administration (oral, transdermal or transvaginal), and multiple answers were permitted. Statistical analyses were performed using SAS ver 9.4 (SAS Institute Inc., Cary, NC, USA).

## RESULTS

The characteristics of the 14,839 women for whom OC use was analyzed are shown in Table 1. The prevalence of OC use in this population was 6.0%. The prevalences of OC use were 2.8% in women who were born in the 1950s, 7.8% in women who were born in the 1960s, 10.6% in women who were born in the 1970s, and 20.8% in women who were born after the 1980s.

**Table 1.** Characteristics of the study population assessed for OC user (*n* = 14,839)

		Number	Proportion (%)
Age at baseline survey, years	20–29	498	3.4
	30–39	5,920	39.8
	40–49	5,698	38.4
	50–59	2,723	18.4
Menstrual status at baseline survey	premenopause	12,227	82.4
	postmenopause	2,116	14.3
	unclear	496	3.3
Menstrual status at the final survey	premenopause	7,104	47.9
	postmenopause	7,139	48.1
	unclear	596	4.0
Body mass index at baseline survey, kg/m^2^	<18.5	1,279	8.6
	≥18.5 and <25.0	11,443	77.1
	≥25.0 and <30.0	1,622	10.9
	≥30.0	276	1.9
	missing	219	1.5
Smoking at baseline survey	no	10,520	70.9
	yes	4,206	28.3
	missing	113	0.8
Alcohol drinking habit at baseline survey	no	10,699	72.1
	yes	3,470	23.4
	missing	670	4.5
Gravidity at baseline survey	0	3,565	24.0
	1	1,574	10.6
	2	3,648	24.6
	3	3,135	21.1
	4	1,547	10.4
	≥5	871	5.9
	missing	499	3.4
Parity at baseline survey	0	4,140	27.9
	1	1,857	12.5
	2	5,183	35.0
	3	2,891	19.5
	≥4	362	2.4
	missing	406	2.7
Hysterectomy at the final survey	yes	1,031	7.0
	no	13,808	93.0
Oophorectomy at the final survey	unilateral	544	3.7
	bilateral	279	1.9
	no	14,016	94.4

**Figure 2.**
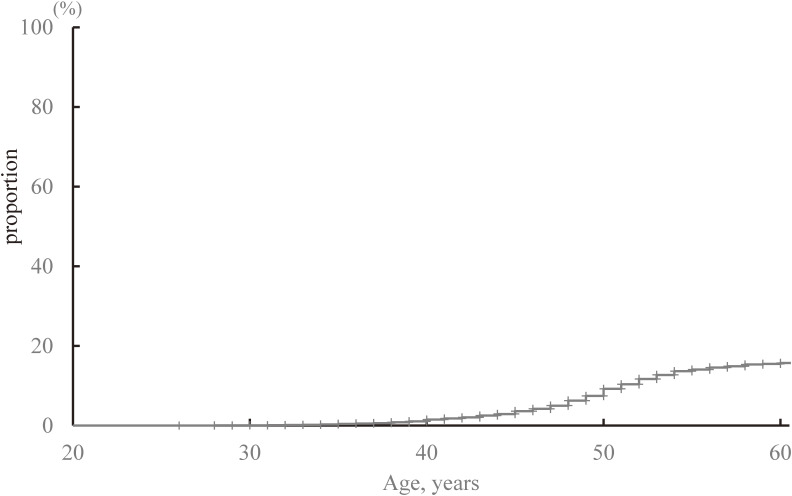
Cumulative incidence of HRT at the age of 60 years estimated using the Kaplan-Meier method

The characteristics of the 7,915 women for whom HRT use was analyzed are shown in Table 2. The proportion of the cohort that had never used HRT was 86.2%. As shown in Table 3, the prevalence of HRT use before the baseline survey (past users) was 3.2% (*n* = 255) and the prevalence of HRT use during the study period (current users) was 10.6% (*n* = 840), making a total prevalence of HRT use of 13.8% (*n* = 1,095). The cumulative incidence of HRT use at the age of 60 years was estimated to be 15.7% by the Kaplan-Meier method (Figure 2).

**Table 2.** Characteristics of the study population assessed for HRT user (*n* = 7,915)

		Number	Proportion (%)
Age at baseline survey, years	20–29	5	0.1
	30–39	649	8.2
	40–49	4,375	55.2
	50–59	2,706	34.2
	≥60s	180	2.3
Menstrual status at baseline survey	premenopause	5,137	64.9
	postmenopause	2,292	29.0
	unclear	486	6.1
Menstrual status at the final survey	postmenopause	7,319	92.5
	unclear	596	7.5
Body mass index at baseline survey, kg/m^2^	<18.5	428	5.4
	≥18.5 and <25.0	6,154	77.7
	≥25.0 and <30.0	1,082	13.7
	≥30.0	147	1.9
	missing	104	1.3
Smoking at baseline survey	no	5,653	71.4
	yes	2,179	27.5
	missing	83	1.1
Alcohol drinking habit at baseline survey	no	5,513	69.7
	yes	2,005	25.3
	missing	397	5.0
Gravidity at baseline survey	0	1,064	13.4
	1	606	7.7
	2	2,162	27.4
	3	2,075	26.2
	4	1,099	13.9
	≥5	645	8.1
	missing	264	3.3
Parity at baseline survey	0	1,255	15.9
	1	861	10.9
	2	3,320	41.9
	3	2,013	25.4
	≥4	271	3.4
	missing	195	2.5
Hysterectomy at the final survey	yes	1,061	13.4
	no	6,854	86.6
Oophorectomy at the final survey	unilateral	435	5.5
	bilateral	287	3.6
	no	7,193	90.9

**Table 3.** HRT users in each category

		Number	Proportion (%)
	Non-users	6,820	86.2
	Users		
	1 Use and cessation before BL	255	3.2
	2 Use before BL and cessation during the study period	262	3.3
	3 Use before BL and at the time of the final survey, but no response to the F10 invitation	16	0.2
	4 Use before BL and at F10	36	0.5
	5 Use after BL and cessation during the study period	364	4.6
	6 Use after BL and at the time of the final survey, but no response to the F10 invitation	18	0.2
	7 Use after BL and at F10	144	1.8

BL, baseline; F10, after the 10-year follow-up study.

The proportions of the women who received HRT were 12.3% in women without hysterectomy and oophorectomy, 13.6% in women with hysterectomy, 15.5% in women with unilateral oophorectomy, 35.9% in women with bilateral oophorectomy, 20.0% in women with unilateral oophorectomy and hysterectomy, and 46.0% in women with bilateral oophorectomy and hysterectomy.

**Figure 3.**
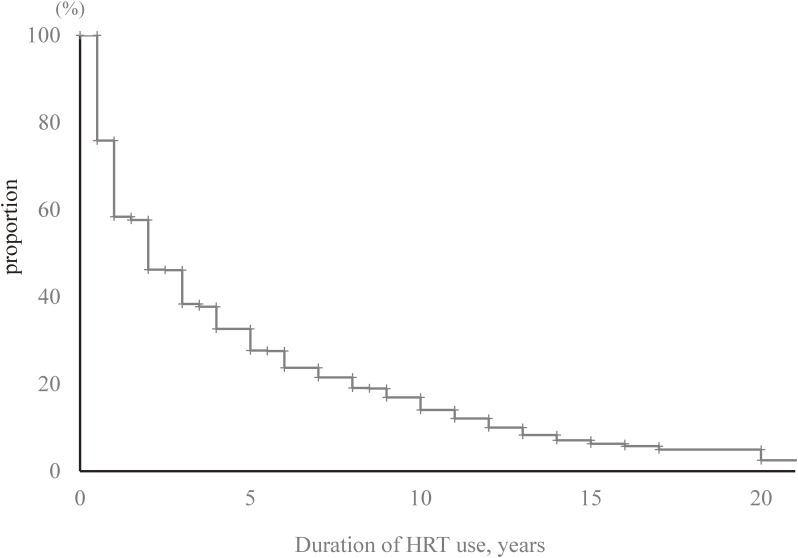
Duration of HRT use estimated using the Kaplan-Meier method

Of the women who had used HRT at any time (*n* = 1,095), 66.2% of the women started HRT between the ages of 45 and 54 years (Table 4). The proportion of women who started HRT at the age of >60 years was 2.1%. The proportions of women who used HRT for 1–3 years, >6 years, and >10 years were 32.7%, 20.2%, and 9.2%, respectively (Table 4). The median duration of HRT use was estimated to be 2 years by the Kaplan-Meier method (interquartile range: 1–5 years; *n* = 1,092 with exclusion of three women whose duration of HRT use was unclear) (Figure 3). With regard to the route of estrogen administration, 65.5% took it orally (717/1,095), 28.8% took it transdermally (315/1,095), and 1.2% took it vaginally (13/1,095). The proportion of women for whom the route of administration was unclear was 12.8% (140/1,095). We counted separately the women who changed the route of administration from oral to transdermal administration.

**Table 4.** Age when commencing HRT and the duration (*n* = 1,095)

		Number	Proportion (%)
Age at the start of HRT, years	<40	84	7.7
	40–44	143	13.1
	45–49	336	30.7
	50–54	389	35.5
	55–59	77	7.0
	≥60	23	2.1
	missing	43	3.9
Duration, years	<1	286	26.1
	≥1 and <3	358	32.7
	≥3 and <6	227	20.7
	≥6 and <10	120	11.0
	≥10	101	9.2
	unclear	3	0.3

## DISCUSSION

We have shown that the lifetime prevalence of OC use for any reason is 6% in female Japanese nurses. Cross-sectional population surveys conducted in 1989–1997 showed that the prevalence of OC use in women aged 34–44 years was >20% in Australia, Belgium, France, and East Germany,^19^ and it was reported that 82% of sexually experienced women used OCs during the period from 2006 to 2008 in the United States.^1^ In the United Kingdom, the prevalence of hormonal contraceptive use among female adolescents increased from 13.7% in 2002 to 19.0% in 2011.^20^ The lifetime prevalence of OC use by Japanese nurses was extremely low compared with those previously reported prevalences. OCs were approved by the Japanese government in 1999. Japan was the last country in the world to approve OCs. The delay in approval of OCs because of the fear about the potential spread of sexually transmitted diseases if OC use replaces condom use might explain the lower popularity of OCs in Japan.^21^ The current use of OC may have changed since about 20 years have passed since OCs were approved in Japan.^22^ We analyzed the prevalences of OC used according to generations, and our results showed that younger women were more likely to use OCs. Most of the participants in the present study were older than women who would most likely use OCs. Therefore, further study of younger women is needed to clarify the use of OCs in Japan in more detail.

In the present study, the lifetime prevalence of HRT use for women who had used HRT and stopped using HRT before the baseline survey was 3.2%, the prevalence of HRT use during the 10-year follow-up period was 10.6%, and the total prevalence of HRT use for peri- and postmenopausal women in the study period was 13.8%. The cumulative incidence of HRT use estimated using the Kaplan-Meier method was 15.7%, which is considered to be closer to the true lifetime prevalence. Considering that the total prevalence could be regarded as being approximately 90% of the cumulative incidence (15.7%) at the age of 60 years estimated by the Kaplan-Meier method, 13.8% is an appropriate value.

There have been few longitudinal studies on the prevalence of HRT use. In the Nurses’ Health Study, it was shown that 15.8% of the participants used HRT during a 14-year period (1980–1994),^13^ and in the Danish Nurse Cohort Study, the prevalence of HRT use between 1993 and 1999 was 37.2%.^14^ It has also been reported that 28.4% of the participants in a cohort study in Denmark used HRT during the period from 1995 to 2010.^15^ The proportion of HRT users in the present study was lower than the proportions in those previous studies. European women start using HRT not only for symptomatic relief but also to reduce the risk of postmenopausal osteoporosis.^23^ In contrast, insurance coverage for the use of estrogen preparations for treatment of osteoporosis is limited in Japan, although the use of estrogen preparations for menopausal symptoms and estrogen-deficiency symptoms is covered by insurance.^24^

The duration of HRT use is an important determinant of its prevalence. The prevalence of HRT use determined in a cross-sectional study may be similar to that in a long-term longitudinal study if the duration of HRT use is long. However, the prevalence of HRT use determined in a cross-sectional survey may be lower if the duration of HRT use is short. We consider that the survey period in the present study in middle-aged and older women was sufficiently long for the prevalence determined in the present study to be close to the true lifetime prevalence.

Du et al^25^ reported that the proportion of long-term HRT users (>3 years) significantly increased from 60.2% in 1997–1999 to 80.1% in 2003–2004. Recently, Løkkegaard et al^15^ reported that the proportion of women who use HRT for 5–9 years is high and that 2–4 years is the next most common duration of HRT use. The International Menopause Society recommended in 2016 that the duration of HRT use should not be limited.^26^ Therefore, the proportion of long-term users may be increasing. In a study conducted in Japan in 1992, it was shown that 9.9% of the women in the survey had used HRT for more than 5 years.^11^ In contrast, in the present study, 20.2% of the women used HRT for more than 6 years. This difference may be explained by differences in the study design since the results obtained in the study conducted in 1992 based on a cross-sectional survey, not a long follow-up survey. In the past 20 years in Japan, there has been not only an increase in the number of women in menopausal transition but also recognition of the effectiveness of HRT and increased awareness by both women and gynecological doctors of menopausal medicine due to the increase in availability of menopausal medicine.

It has been reported that benefits of HRT are likely to outweigh the risks if symptomatic women start HRT before the age of 60 years or within 10 years after menopause.^7^ The results of the present study showed that 66.2% of the women aged 45–54 years had started HRT, and that percentage is close to the percentages reported in the United States and European countries.^27^^,^^28^ It was reported that the proportion of women who started HRT at more than 54 years of age in the period from 1990 to 2001 was 16.8%,^29^ and that the proportion of women who started HRT at more than 55 years of age in a survey conducted in 2003 was 9%.^23^ Consistent with those reports, the percentage of women who started HRT at more than 55 years of age in the present study was 9.1%.

In the present study, the prevalence of HRT use in women who had undergone bilateral oophorectomy was high, although the prevalence of HRT use in women who had undergone hysterectomy was similar to that in women who had not undergone hysterectomy and oophorectomy. Further detailed study on HRT prevalence in women who have undergone bilateral oophorectomy is needed.

Because the JNHS was a long-term study, the prevalence of HRT use determined in the present study is considered to be close to the true lifetime prevalence, and that is a strength of the present study. However, this study has several limitations. First, we asked about OCs as exogenous hormones other than those used in HRT and did not ask about age of starting and duration of taking OCs in detail. Since there have been changes in social situations, such as the addition of insurance coverage for treatment of endometriosis by OCs in 2008 and the introduction of low-dose estrogen and progestin, it is difficult to determine whether the drugs taken by women during the 10-year period in this study were actually OCs. Second, the results of the present study may not be applicable to women in general, since the subjects of this study were nurses, who have easier access to medications. Third, we could not obtain information on the precise dates of stopping and resuming HRT use, but we had asked year of age when they started and stopped HRT use in the biennial follow-up surveys. We considered a duration of HRT use for less than 1 year as 0.5 years and took into account HRT users whose duration of use was less than 1 year in Kaplan-Meier calculations. Thus, this may not affect the results of this study.

In conclusion, the lifetime prevalences of OC use and HRT use were 6.0% and 13.8%, respectively, in Japanese nurses in this prospective study. The results of this study will be useful in future research clarifying the association between exogenous estrogen exposure and estrogen-related diseases.
